# A Comparative Study of New* Aspergillus* Strains for Proteolytic Enzymes Production by Solid State Fermentation

**DOI:** 10.1155/2016/3016149

**Published:** 2016-02-16

**Authors:** Gastón Ezequiel Ortiz, Diego Gabriel Noseda, María Clara Ponce Mora, Matías Nicolás Recupero, Martín Blasco, Edgardo Albertó

**Affiliations:** Instituto de Investigaciones Biotecnológicas-Instituto Tecnológico de Chascomús (IIB-INTECH), Universidad Nacional de San Martín (UNSAM) and Consejo Nacional de Investigaciones Científicas y Técnicas (CONICET), San Martín, 1650 Buenos Aires, Argentina

## Abstract

A comparative study of the proteolytic enzymes production using twelve* Aspergillus* strains previously unused for this purpose was performed by solid state fermentation. A semiquantitative and quantitative evaluation of proteolytic activity were carried out using crude enzymatic extracts obtained from the fermentation cultures, finding seven strains with high and intermediate level of protease activity. Biochemical, thermodynamics, and kinetics features such as optimum pH and temperature values, thermal stability, activation energy (*E*_a_), quotient energy (*Q*_10_), *K*_*m*_, and *V*_max_ were studied in four enzymatic extracts from the selected strains that showed the highest productivity. Additionally, these strains were evaluated by zymogram analysis obtaining protease profiles with a wide range of molecular weight for each sample. From these four strains with the highest productivity, the proteolytic extract of* A. sojae* ATCC 20235 was shown to be an appropriate biocatalyst for hydrolysis of casein and gelatin substrates, increasing its antioxidant activities in 35% and 125%, respectively.

## 1. Introduction

Proteases constitute a large and complex group of hydrolytic enzymes with important applications in medical, pharmaceutical, biotechnology, leather, detergent, and food industries [1]. They can be synthesized by plants, animals, and microorganisms constituting around 60% of the worldwide enzyme market [2]. Among such sources, microorganisms present remarkable potential for proteolytic enzymes production due to their extensive biochemical diversity and susceptibility to genetic manipulation [3]. Filamentous fungi have been utilized for the production of diverse industrial enzymes because these organisms exhibit the capacity to grow on solid substrates and secrete a wide range of hydrolyzing enzymes. Particularly, several species of* Aspergillus* have been exploited as important sources of extracellular enzymes including proteases [4]. According to the state of the art, most of the current scientific knowledge associated with the proteases production by* Aspergillus* fungus is related to the use of* A. oryzae* and* A. niger* species (Supplementary Data 1 in Supplementary Material available online at http://dx.doi.org/10.1155/2016/3016149). Products of* Aspergillus* species such as* A. niger*,* A. sojae*, and* A. oryzae* have acquired a Generally Recognized as Safe (GRAS) status from the US Food and Drug Administration, which has approved their use in the food industry [5].

The production of proteases can be performed by solid state fermentation (SSF) and submerged fermentation (SmF) [6]. The application of SSF is of interest for fungi enzymes production due to its advantages in comparison to SmF, such as low fermentation technology, low cost, higher yields and concentration of the enzymes, and reduced waste output [7, 8]. Furthermore, inexpensive and widely available agricultural solid wastes could be used with the aim of providing nutritional and physical support throughout SSF procedures [9]. The intense demand for industrial proteolytic enzymes requires the search of new strains with high level of protease productivity in order to enhance the enzyme production capacity and their applications [2]. In such context, the aim of this work is to expand the range of species and strains of the genus* Aspergillus* suitable for the production of proteolytic enzymes under solid state fermentation for industrial use.

## 2. Materials and Methods

### 2.1. State of the Art Analysis

The state of the art for proteases production by* Aspergillus* was evaluated through a literature search in the Scopus and PubMed database using the following criteria of search: TITLE ((*Aspergillus* AND Proteases) and Production). The publications nondirectly related to the proteases production were dismissed (Supplementary Data 1).

### 2.2. Microbial Strains

The* Aspergillus* strains used in this work were (1)* A. terreus* ICFC 744/11; (2)* A. oryzae* NRRL 2217; (3)* A. awamori* NRRL 356; (4)* A. flavipes* NRRL 295; (5)* A. kawachii* IFO 4308; (6)* Aspergillus* sp. ICFC 7/14; (7)* A. japonicus* NRRL 1782; (8)* A. oryzae* ICFC 8/12; (9)* A. giganteus* NRRL 10; (10)* A. rhizopodus* NRRL 6136; (11)* A. sojae* NRRL 5595; and (12)* A. sojae* ATCC 20235. Such strains are conserved in the IIB-INTECH Collection of Fungal Cultures (ICFC), reference in the WDCM database: WDCM 826. All the strains were periodically propagated and maintained on potato dextrose agar slants.

### 2.3. Inoculum Preparation

In order to produce conidia for inoculation of the main cultures, the strains were grown on agar-plates containing sugarcane molasses (45 g/L), peptone (18 g/L), NaCl (5 g/L), KCl (0.5 g/L), FeSO_4_·7H_2_O (15 mg/L), KH_2_PO_4_ (60 mg/L), MgSO_4_ (50 mg/L), CuSO_4_·5H_2_O (15 mg/L), MnSO_4_ (15 mg/L), and agar (20 g/L). Plates were incubated at 28°C until sporulation. Conidia were harvested from the plates by the addition of 5 mL of 0.08% (w/v) Tween80. The number of conidia/mL in the conidia suspension was determined using Neubauer cell-counting chamber.

### 2.4. Culture Conditions

Erlenmeyer flasks (250 mL) containing 10 g of wheat bran with a homogeneous particle size of 2000 *μ*m in average were moistened at 110% with Czapek-Dox medium corresponding to 0.96 units of water activity (Supplementary Data 2). The flasks with the sterile culture substrate (sterilization conditions, 121°C for 20 min) were inoculated with 10^6^ conidia/g of dry substrate (gds) and incubated at 28°C in a moist chamber for 2, 4, and 6 days.

### 2.5. Enzyme Recovery

Enzymes produced were recovered by the addition of 10 mL/gds of distilled water into each culture flask and mixing in a shaker at 250 rpm, 28°C, for 30 min. Then, the mixtures were clarified by filtration through cotton followed by centrifugation at 2000 ×g, 4°C, for 20 min. The clarified supernatants were used for the following analysis.

### 2.6. Semiquantitative Determination of Proteolytic Activity by Agar-Plate Assay

A semiquantitative determination of proteolytic activity was carried out at different values of pH by the agar-plate diffusion assay according to the technique described by Heerd et al. [5]. Agar-plates were prepared with 1.5% (w/v) agar and 1.5% (w/v) skim milk as substrate. Both agar and skim milk were dissolved in 0.1 M Tris-HCl buffer in order to adjust and maintain the pH between 6 and 9. Wells of 4 mm diameter were punched in the solid media and loaded with 20 *μ*L crude enzymatic extracts. After 18 h incubation at 30°C, the milk agar-plates were stained with Coomassie Brilliant Blue G-250 for 20 min. The diameter of the halos (*D*) corresponding to the zone of milk degradation was converted to log_10_⁡ by (1) and reported as hydrolysis index (log_10_⁡ mm^2^) [5]:

(1) log10⁡ adjusted  zone  area=log10⁡D22π−4.022π.

Semiquantitative determination was performed by means of 2 independent assays. Proteolytic activity values from different crude extracts were evaluated by Multifactorial ANOVA and cluster analysis using the statistical software Statgraphics Centurion XVII trial version (Supplementary Data 3).

### 2.7. Quantitative Analysis of Proteolytic Activity

Protease activity was quantitatively measured using azo-casein assay according to Cavello et al. [10], with modifications. Reaction mixture containing 20 *μ*L of enzyme extract diluted in 0.1 M Tris-HCl buffer (pH: 8) (buffer T) and 50 *μ*L of 1% (w/v) azo-casein solution in buffer T was incubated for 60 min at 37°C in thermocycler machine. Reaction was stopped with the addition of 100 *μ*L of 10% (v/v) trichloroacetic acid. The mixture was kept at room temperature for 15 min and then centrifuged at 2000 ×g, 20°C, for 10 min. Finally, 50 *μ*L of each sample was diluted by addition of 50 *μ*L of 1 M NaOH solution and absorbance was measured at 415 nm with a microplate spectrophotometer. All determinations were performed in duplicate and a heat-inactivated enzyme extract was used for blank. One unit of proteolytic activity (U) was defined as the amount of enzyme that produces an increase of 0.1 units in the absorbance at 415 nm per min under test conditions. Azo-casein was synthesized as described by Riffel et al. [11].

### 2.8. Effect of Temperature and pH on Proteolytic Activity

The optimal pH and temperature for crude enzymatic extracts were determined employing a central composite design (CCD) with four axial points and three central points. In order to maximize the variability, the experiments were randomized and performed as shown in Supplementary Data 4. Enzymatic extracts corresponding to 2 days of fermentation were normalized to 30 U/mL and incubated at different temperature and pH values during 1 h. For this, the enzymatic extracts were diluted with 0.1 M maleate buffer or 0.1 M Tris-HCl to adjust the pH to lower or higher values than 7.0, respectively. The protease activity was determined using the azo-casein method and the second-order model represented by (2) was used to describe this response:

(2) $$\begin{array}{c} Y=\beta_{0}+\overset{n}{\underset{i=1}{\sum }}\beta_{i}x_{i}+\overset{n-1}{\underset{i=1}{\sum }}\;\overset{n}{\underset{j=i+1}{\sum }}\beta_{ij}x_{i}x_{j}, \end{array}$$

where *Y* is the estimated response, *β*_0_ is the constant term, *i* and *j* have values from 1 to the number of variables (*n*), *β*_*i*_ is the linear coefficient, *β*_*ij*_ is the quadratic coefficient, and *x*_*i*_ and *x*_*j*_ are the coded independent variables. The coefficient of determination *R* ^2^ and the *F* value from analysis of variance (ANOVA) were used to confirm the quality of the model. Relationships between the responses and variables were evaluated using Statgraphics Centurion XVII software trial version.

### 2.9. Enzymatic Stability

The proteolytic stability was analyzed by incubating the crude extracts under optimal pH and temperature values at different periods of time before conducting enzymatic activity determination by azo-casein method. Due to the complexity of the reaction occurring during inactivation, several equations have been proposed to model this kinetic. In this work, a first-order kinetic model (3) was selected to represent the residual enzyme activity (*A*/*A*_0_) at time (*t*, min). The parameter *k* (min^−1^) is the rate constant of the reaction under assay conditions:

(3) $$\begin{array}{c} \frac{A}{A_{0}}=e^{-kt}. \end{array}$$

### 2.10. SDS-PAGE and Zymogram Analysis

SDS-PAGE electrophoresis was performed according to the technique described by Laemmli [12]. The enzyme extracts were diluted in loading buffer without DDT to a final concentration of 10 U/mL. A volume of 20 *μ*L of each sample was loaded on a 10% (v/w) separating SDS-PAGE gel by duplicate in symmetric disposition. The electrophoresis was conducted at 160 V and 4°C during 1 h. After that, the gel was cut in halves, keeping one for the zymogram and staining the other with colloidal Coomassie Brilliant Blue G-250 [13]. Zymography analysis was performed according to Cavello et al. [14] with slight modifications. Briefly, the gel was submerged in 100 mM Tris-HCl buffer (pH 8.0) (buffer T) containing 2.5% Triton X-100 during 60 min, with constant agitation, and then washed three times with buffer T. Finally, the gel was incubated with 1% (w/v) casein in buffer T at 30°C for 60 min and then stained with Coomassie Brilliant Blue R-250. The development of clear bands on the blue background of the gel indicated the presence of protease activity. The molecular weight of proteolytic enzymes was estimated through densitometry analysis using Image J software 1.44p version [15].

### 2.11. Determination of Activation Energy and Temperature Quotient (*Q*_10_)

Activation energy (*E*_a_) values of the proteases produced by* Aspergillus* strains were calculated by incubating enzyme extracts, under optimal pH condition, with 1% azo-casein at several temperatures ranging from 30 to 50°C. The dependence of the rate constants with temperature was assumed to follow Arrhenius Law (4) and *E*_a_ was calculated from the slope of the plot of 1000/*T* versus ln (protease activity), where *E*_a_ = −slope × *R*, *R* (gas constant) = 8.314 (J/K·mol), and *T* is the absolute temperature in Kelvin (K) [16]. The temperature quotient (*Q*_10_) values of* Aspergillus* proteases were determined using (5) [17]:

(4) ln⁡Activity=ln⁡A−EaR×1T,

(5) Q10=antilog×e−Ea×10/RT2.

### 2.12. Kinetics Parameters *K*_*m*_ and *V*_max_

Kinetics parameters *K*_*m*_ and *V*_max_ of the proteases produced by* Aspergillus* strains were determined by incubating crude extracts, under optimal pH and temperature values, with azo-casein over the concentration range 1.0–10.0 mg/mL. Protease activity of each extract was quantitatively measured as described in Section 2.7. The Michaelis-Menten constant (*K*_*m*_) and maximum velocity (*V*_max_) values were calculated using the GraphPad Prism 4® trial version. The values employed in the nonlinear regression are shown in Supplementary Data 7.

### 2.13. Preparation of Protein Hydrolysates

For the production of protein hydrolysates, the enzyme concentration of a selected crude extract was adjusted to 400 U per gram of dry substrate (U/gds) according to its proteolytic activity established previously. Gelatin and casein substrates were suspended in buffer citrate phosphate 0.1 M to a final concentration of 1.0% (w/v). A total of 15 mL of each mixture was distributed in 50 mL Erlenmeyer flasks and incubated in a water bath shaker operating at 50 rpm. The hydrolysis was performed at the optimal temperature and pH values of the enzyme extract during 120 min. Aliquots (0.5 mL) of each mixture were taken at regular intervals and inactivated in a water bath at 100°C for 15 min. A volume of 0.1 mL was reserved to determine the degree of hydrolysis. The remaining volume (0.4 mL) was centrifuged at 12,500 ×g in Eppendorf MiniSpin® for 10 min for separating the soluble peptides from nonhydrolyzed proteins. The supernatants were collected to determine the antioxidant activities.

### 2.14. Determination of Antioxidant Activities

The antioxidant activity of the protein hydrolysates was determined by the DPPH radical-scavenging method as described by Bougatef et al. [18] with adaptations for microplate assay. A volume of 50 *μ*L of the diluted hydrolysates was mixed with 50 *μ*L of 99.5% ethanol and 30 *μ*L of 0.02% DPPH in 99.5% ethanol. The mixtures were kept at room temperature in the dark for 60 min, and the reduction of the DPPH radical was measured at 540 nm using a microplate reader Bio-Rad Benchmark®. The DPPH radical-scavenging activity was calculated using

(6) Radical  scavening  activity%=Control  Abs540 nm−Sample  Abs540 nmControl  Abs540 nm×100%.

### 2.15. Determination of the Degree of Hydrolysis

The degree of hydrolysis of the protein hydrolysates was determined according to the method described by Adler-Nissen [19]. Mixtures of 0.5 mL of diluted hydrolysates or 0.825 mmol/L standard cysteine amino acid and 0.250 mL of 0.01% (w/v) TNBSA in 0.1 M sodium bicarbonate pH 8.5 were incubated for 30 min at 37°C. A volume of 200 *μ*L of each mixture was taken and the absorbance measured at 415 nm in a Bio-Rad Benchmark microplate reader. Degree of hydrolysis (DH) values were calculated using

(7) Cysteine-NH2meqvg=X×V×f×Sample  Abs415 nm−Blank  Abs415 nmStandard  Abs415 nm−Blank  Abs415 nm×0.82 meqv/L,h=Cysteine-NH2−βα,DH%=hhtot×100%,

where cysteine-NH_2_ is milliequivalents of cysteine amine groups per gram of protein; *X* is mass of sample protein in gram (g); *V* is the reaction volume in liter (L); and *f* is dilution factor. The value *h* is the number of hydrolyzed peptide bonds and *h*_tot_ is the total number of peptide bonds per protein equivalent. The values *α*, *β*, and *h*_tot_ for casein and gelatin are *α* = 1.039, *β* = 0.383, and *h*_tot_ = 8.2 and *α* = 0.796, *β* = 0.457, and *h*_tot_ = 11.1, respectively [20].

## 3. Results and Discussion

### 3.1. Semiquantitative Determination of Proteolytic Activity

**Figure 1:**
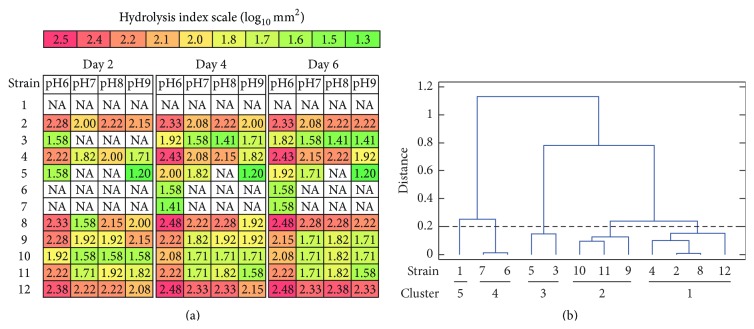
Semiquantitative analysis of proteolytic activity from* Aspergillus* spp. extracts. Protease activity was semiquantitatively analyzed for different values of pH from crude extracts of* Aspergillus* ssp. cultures obtained by solid state fermentation. (a) Proteolytic activity values expressed by the hydrolysis index (log_10_⁡ mm^2^). The values correspond to the average of two independent studies. NA: no activity. (b) Dendrogram constructed from the proteolytic activities using median method and squared Euclidean distance. Distance of 0.2 indicates the cut-line for cluster designation. For strain reference numbers see Section 2.2.

To evaluate the capability of* Aspergillus* strains for proteases production, crude enzymatic extracts obtained by SSF were semiquantitatively analyzed for total proteolytic activity at different pH values by agar-plate diffusion assay. In this study the diameters of proteolysis halos were adjusted as indicated in Section 2.6 to report the enzymatic activity index. Such values were employed to compare the strains through multifactorial analysis of variance (ANOVA) (Supplementary Data 3). Wheat bran was chosen as the screening substrate because it possesses a suitable carbon to nitrogen ratio (C : N) and water absorption index, necessary for an appropriate growth and protease production as was reported by Soares de Castro et al. [21, 22]. Analysis of variance revealed that most of the evaluated strains registered significantly higher proteolytic activity at days 4 and 6 of fermentation and with a pH value of 6.0 (Figure 1(a)). Furthermore, the cluster analysis (Figure 1(b)) revealed the presence of five homogeneous clusters of strains based on its enzymatic activity. Cluster 1, which included* A. sojae* ATCC 20235,* A. oryzae* ICFC 8/12,* A. oryzae* NRRL 2217, and* A. flavipes* NRRL 295, showed a high protease activity throughout the tested pH range, while cluster 2, formed by* A. giganteus* NRRL 10,* A. sojae* NRRL 5595, and* A. rhizopodus* NRRL 6136, presented intermediate values of proteolytic activity in the pH range 7.0–9.0, displaying an increase in the activity at pH 6.0. Furthermore, cluster 3 (*A. kawachii* IFO 4308 and* A. awamori* NRRL 356 strains) exhibited a reduced proteolytic activity throughout the pH range for days 4 and 6. Cluster 4 (*Aspergillus* sp. ICFC 7/14 and* A. japonicus* NRRL 1782 strains) showed very low proteolytic activity at pH 6.0 on days 4 and 6. Finally, cluster 5 did not record proteolytic activity. Based on these results it can be concluded that crude extracts from strains of cluster 1 provided the highest level of protease activity. It should be mentioned that the strains of such cluster,* A. flavipes* NRRL 295 and* A. sojae* ATCC 20235, do not possess scientific reports for proteolytic enzymes production. However, it is well known that* A. sojae* and* A. oryzae* are employed industrially for commercial production of proteases [2]. On the other hand, the* A. sojae* NRRL 5595,* A. giganteus* NRRL 10, and* A. rhizopodus* NRRL 6136 strains (cluster 2) provided intermediate level of proteolytic activity. It is important to highlight that the last two species were not previously reported for protease production.

### 3.2. Proteolytic Activity and Productivity Analysis

**Figure 2:**
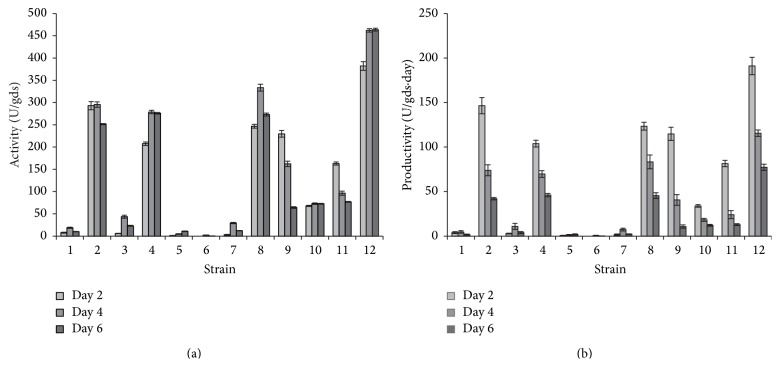
Protease production and productivity from* Aspergillus* spp. extracts. (a) Protease production expressed as proteolytic activity for 3 different periods of culture. (b) Proteolytic productivity during fermentation process. Experiments were performed in triplicate and error bars represent the standard deviation. For strain reference numbers see Section 2.2.

In order to validate the results achieved previously a quantitative study of the proteolytic activity and enzymatic productivity was conducted. As shown in Figure 2(a) the strains belonging to cluster 1 (Figure 1(b)) presented the highest level of proteolytic activity. In this cluster, the* A. sojae* ATCC 20235 and* A. flavipes* NRRL 295 showed a similar protease production pattern in which the activity increased and remained constant during the culture suggesting an adequate stability of the crude extracts, whereas* A. oryzae* ICFC 8/12 and* A. oryzae* NRRL 2217 exhibited a decrease in the proteolytic activity on day 6 of incubation, which suggested certain instability of these enzymes. Furthermore,* A. giganteus* NRRL 10,* A. rhizopodus* NRRL 6136, and* A. sojae* NRRL 5595 that formed cluster 2 (Figure 1(b)) showed a lower enzyme activity which decreased over the culture course for* A. giganteus* NRRL 10 and* A. sojae* NRRL 5595, but remained stable with a low value for* A. rhizopodus* NRRL 6136 suggesting an appropriate enzymatic stability for this strain. On the other hand, the protease production profile observed for* A. giganteus* NRRL 10,* A. rhizopodus* NRRL 6136, and* A. sojae* NRRL 5595 is similar to the production profile reported by Soares de Castro et al. for* A. oryzae* LBA01 and* A. niger* LB02, two species well known for their good productivity of proteolytic enzymes [3, 23]. In addition, maximum protease productivity was registered for day 2 of fermentation for strains of clusters 1 and 2 (Figures 2(b) and 1(b)). These results confirmed that strains from cluster 1 presented the highest level of protease production and demonstrated that maximum productivity was achieved after a short fermentation time. On the other hand, and according to the results obtained with the semiquantitative analyses, crude extracts from the strains of the clusters 3, 4, and 5 showed the lowest proteolytic activity (Figures 2(a) and 1(b)) and productivity (Figure 2(b)).

### 3.3. Influence of Temperature and pH on Proteolytic Activity

**Figure 3:**
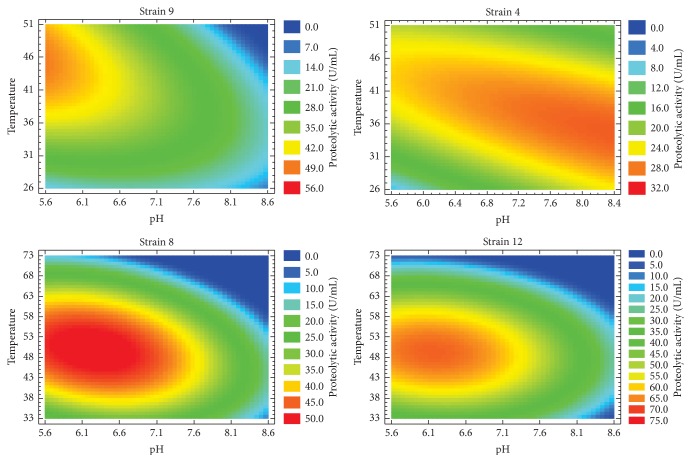
Effect of pH and temperature on proteolytic activity. Enzyme activities analyzed through central composite design. The dependence of the activity with pH and temperature is represented as a contour plot. Red and blue colors indicate the values of maximum and minimum activities, respectively. For strain reference numbers see Section 2.2.

The optimal temperature of fungal proteases ranged between 35 and 50°C with few exceptions and the optimum pH values for acid and alkaline proteolytic enzymes range between 2.0–6.0 and 8.0–11.0, respectively, while optimal pH for neutral proteases and metalloproteases ranges from 6 to 8 [2]. Therefore, in order to determine the optimum pH and temperature of proteases from the strains with higher proteolytic productivity,* A. flavipes* NRRL 295,* A. oryzae* ICFC 8/12,* A. giganteus* NRRL 10, and* A. sojae* ATCC 20235, we performed a central composite design with four axial points as was described in Section 2.8 and the results were analyzed by ANOVA to confirm the quality of each model and the adequacy of these models was validated by three verification trials (Supplementary Data 4). It should be mentioned that* A. oryzae* ICFC 8/12 was selected as a control strain for this analysis since it is a well-studied strain. As shown in Figure 3, enzymatic extracts from strains* A. oryzae* ICFC 8/12 and* A. sojae* ATCC 20235 presented maximum proteolytic activity in a pH range of 6.0 to 6.8 and a temperature range between 43 and 53°C, suggesting that these extracts would be composed mainly of neutral and thermophilic proteases. In this sense, the production of neutral proteolytic enzymes had previously been reported for* A. oryzae* NRRL 2220 and* A. sydowii* [24, 25]. Furthermore, we determined that* A. flavipes* NRRL 295 had maximum activity around pH 8.4 and in the temperature range 32–43°C, suggesting the presence of alkaline and mesophilic proteases in such extract. In this respect, proteases with these biochemical features were previously described for* A. tamarii* [26]. Meanwhile,* A. giganteus* NRRL 10 exhibited maximum activity around pH 5.6 and with a temperature range 41–51°C, indicating that the extract would be mainly composed of acidic and thermophilic proteases. In this sense, the production of enzymes with similar characteristics has previously been reported for* A. oryzae* LBA01 and MTCC 5341 [23, 27]. In accordance with the results obtained from the semiquantitative analysis (Figure 1),* A. sojae* ATCC 20235,* A. oryzae* ICFC 8/12, and* A. flavipes* NRRL 295 showed proteolytic activity over the broad pH range 5.6–8.4, while* A. giganteus* NRRL 10 presented higher activity at acidic pH values.

### 3.4. Thermal and pH Stability of* Aspergillus* Proteases

**Figure 4:**
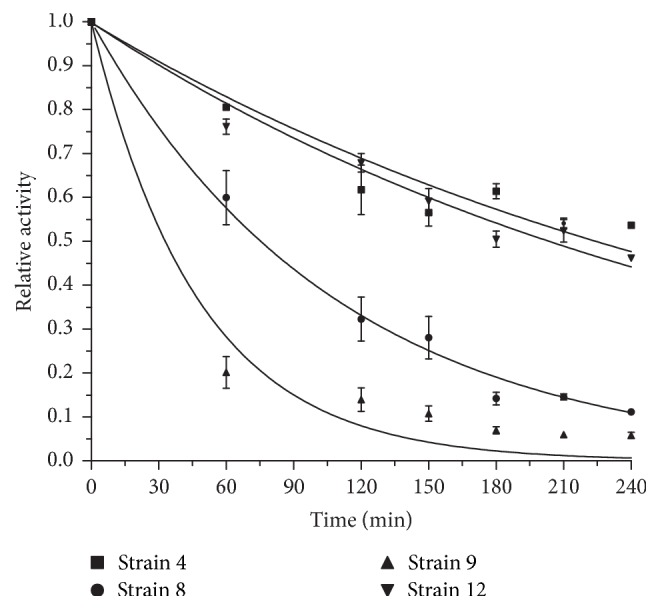
Thermal and pH stability study of protease extracts. Fraction of enzymes remaining active after incubation under optimal conditions. Three independent samples were assayed for each enzymatic extract and error bars represent the standard deviation.

The stability of enzymes appears as an important critical aspect to conduct industrial application. Therefore, thermal and pH stability of proteolytic extracts were evaluated under the optimal conditions previously determined.  Figure 4 and Table 3 show the decay curves of proteolytic activity for the evaluated enzymatic extracts. The extracts from* A. sojae* ATCC 20235 and* A. flavipes*, tested under pH 6.4 and 48°C and pH 8.4 and 36°C, respectively, presented similar stability with an inactivation constant of 0.003 min^−1^ and a half-life time of around 215 min. These enzymes resulted as more stable than other proteases previously reported for* Aspergillus* species such us* A. clavatus* strains ES1 (half-life: 30 min at 50°C) and CCT2759 (half-life: 18 min at 50°C) [28]. On the other hand, the extract from* A. giganteus*, analyzed at pH 5.6 and 48°C showed a high inactivation constant of 0.021 min^−1^ and a half-life of about 33 min reflecting poor level of stability. Proteases from* Penicillium* sp. showed similar stability properties with a half-life of 30 min at 45°C [29]. Meanwhile,* A. oryzae* ICFC 8/12 which was evaluated under pH 6.4 and 48°C exhibited an intermediate stability with an inactivation constant of 0.009 min^−1^ and a half-life time of 75 min.

### 3.5. Electrophoresis SDS-PAGE and Zymogram Analysis

**Figure 5:**
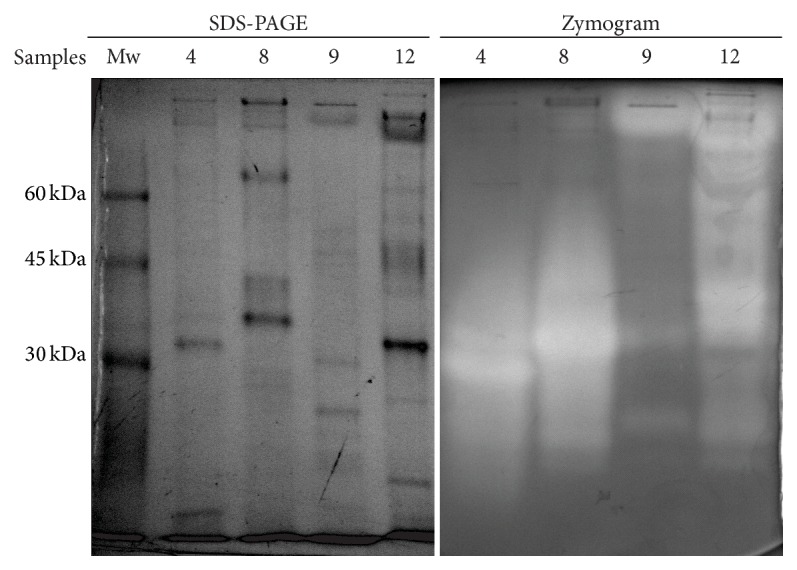
Analysis of crude extracts by SDS-PAGE electrophoresis and zymogram. Samples: Mw: protein molecular weight marker; 4:* A. flavipes* NRRL 295; 8:* A. oryzae* ICFC 8/12; 9:* A. giganteus* NRRL; 10, 12:* A. sojae* ATCC 20235.

The molecular weights of fungal proteases are generally in the range of 20 and 50 kDa [2], with some exception such as low-molecular weight alkaline protease (6.8 kDa) from* Conidiobolus coronatus* [30] and high-molecular weight thiol proteinase (237 kDa) from* Humicola lanuginosa* UAMH 1623 [31]. Likewise, the diversity in molecular weights of fungal proteases has been used to differentiate the species* A. sojae* and* A. oryzae* on the basis of specific mobility of alkaline proteases in polyacrylamide gel disc electrophoresis [32]. In order to evaluate the total protein pattern and the taxonomic proteolytic enzyme profile, crude enzymatic extracts corresponding to strains* A. sojae* ATCC 20235,* A. oryzae* ICFC 8/12,* A. giganteus* NRRL 10, and* A. flavipes* NRRL 295 were analyzed under alkaline condition by polyacrylamide gel electrophoresis and zymogram. Hence, SDS-PAGE analysis (Figure 5) allowed us to distinguish a clear difference between the protein patterns of the four examined strains. Likewise, such patterns presented remarkable bands throughout the whole range of molecular weight (100–10 kDa). Furthermore, as shown in Table 4,* A. flavipes* NRRL 295 and* A. oryzae* ICFC 8/12 strains exhibited bands with high proteolytic activity in the range 20–35 kDa. These results are in accordance with previous studies that reported an alkaline serine-protease from* A. clavatus* ES1 with a molecular mass of 32 kDa and an alkaline protease from* A. terreus* with a molecular weight of 37 kDa [33, 34]. In addition, crude extracts from the strains of* A. giganteus* and* A. sojae* presented remarkable active bands with molecular masses in the range 85–95 kDa which was consistent with a high-molecular mass protease (124 kDa) from* A. fumigatus* TKU003 reported by Wang et al. [35]. Chien and coworkers reported the purification, characterization, and cloning of a leucine aminopeptidase (LAP) from* A. sojae* with an apparent molecular mass of 37 kDa [36]. In this sense, a protease with a similar relative molecular weight could be produced by* A. sojae* ATCC 20235 (Table 4).

### 3.6. Activation Energy and Temperature Quotient

The activation energies of the proteases produced by* Aspergillus* strains were determined at the temperature ranges that are indicated in Table 1. The Arrhenius plots for such proteases showed a linear variation with temperature increase, suggesting that these proteolytic enzymes have single conformations up to the transition temperatures (Supplementary Data 5) [16]. As shown in Table 1, the minimal activation energy (21.82 kJ/mol) necessary to conduct the hydrolysis of azo-casein was obtained for alkaline proteases from* A. flavipes* NRRL 295. Likewise, the intermediate energies values were obtained for acid proteases from* A. sojae* ATCC 20235 and* A. giganteus* NRRL 10. On the other hand* A. oryzae* ICFC 8/12 presented the highest activation energy (38.64 kJ/mol). In this context, Melikoglu and coworkers [16] reported an activation energy of the 36.8 kJ/mol for bread protein hydrolysis employing proteases from* A. awamori* in a temperature range of 30–55°C. This value was similar to those obtained for the strains of* A. sojae*,* A. giganteus*, and* A. oryzae*. On the other hand proteolytic extracts from* A. niger* in a range of 35–50°C exhibited activation energies values for azo-casein hydrolysis that ranged from 16.32 to 19.48 kJ/mol [17]. These values are similar to that obtained from the strain of* A. flavipes*. To study the effect of temperature on the rate of reaction, we investigated the temperature quotient (*Q*_10_), that is, a measure of the rate of change of a biological or chemical system as a consequence of increasing the temperature by 10°C. The *Q*_10_ values of the examined extracts ranged from 1.31 to 1.61 (Table 1). Generally enzymatic reactions show *Q*_10_ values between 1.00 and 2.00 units and any deviation from this value is indicative of involvement of some factor other than temperature in controlling the rate of reaction. Soares de Castro and coworkers reported the *Q*_10_ values between 1.20 and 1.28 for azo-casein hydrolysis with temperatures between 30 and 60°C [17]. The maximum value of *Q*_10_ was obtained for proteases from* A. oryzae* ICFC 8/12 reflecting that for every 10°C raise in temperature the rate of reaction increased 61%.

### 3.7. Kinetic Parameters *K*_*m*_ and *V*_max_

In order to evaluate the kinetics properties of proteolytic enzymes, the parameters *V*_max_, *K*_*m*_, and *V*_max_/*K*_*m*_ were calculated for each proteases extract under optimal pH and temperature conditions (Table 2). The maximum catalysis velocity (*V*_max_) is the amount of enzyme involve in the enzymatic reaction. The *K*_*m*_ parameter provides the affinity of enzyme for substrate: a low value of this parameter indicates a higher affinity enzyme-substrate. The ratio *V*_max_/*K*_*m*_ is related to the specificity and efficiency of enzymes: a high value of this parameter indicates a higher catalytic specificity and efficiency [37, 38].

The highest affinity for azo-casein was observed for proteolytic extract from* A. oryzae* ICFC 8/12 with a *K*_*m*_ value expected at 0.86 mg/mL, followed by proteases from* A. flavipes* NRRL 295,* A. sojae* ATCC 20235, and* A. giganteus* NRRL 10 (Table 2). A wide *K*_*m*_ value for azo-casein hydrolysis has been reported. Li and coworkers reported a *K*_*m*_ value of 0.96 mg/mL for an acid protease from a* A. oryzae* and* A. niger* fusant strain [4]. Furthermore, Soares de Castro et al. reported a wide range of *K*_*m*_ (0.44–1.92 mg/mL) when* A. niger* was grown on different substrates [17]. Murthy and Naidu reported a higher *K*_*m*_ value (3 mg/mL) for an alkaline protease from* A. oryzae* growing in solid state fermentation using coffee waste as substrate [39]. The *V*_max_ values of the proteolytic extracts ranged between 44.6 U/gds and 233.8 U/gds (Table 2). Similar range values were reported by Soares de Castro et al. for* A. niger* grown on different substrates [17]. The highest substrate specificity and catalytic efficiency (*V*_max_/*K*_*m*_ ratio) was achieved for proteases from* A. oryzae* ICFC 8/12 and* A. sojae* ATCC 20235 (Table 2); similar values were obtained for proteases from* A. niger* grown on wheat bran [17].

### 3.8. Hydrolysis Degree and Antioxidant Activity

**Figure 6:**
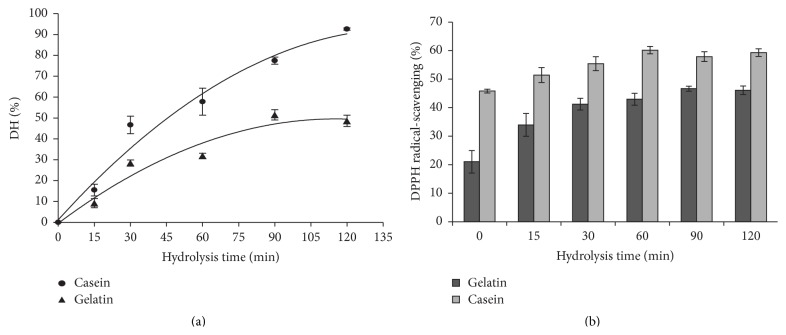
Hydrolysis and antioxidant activity assay. (a) Degree of hydrolysis (DH) for casein and gelatin substrates using proteases from* A. Sojae* ATCC 20235. (b) DPPH radical-scavenging activity using the hydrolysates from casein and gelatin. The error bars correspond to three independent assays.

Proteases from different* A. oryzae* strains have been used in the hydrolysis of whey protein or gelatin with the purpose of obtaining functional peptides with antioxidant activity [40, 41]. In this context and considering that* A. sojae* ATCC 20235 achieved the highest productivity and exhibited similar biochemical characteristics (Figure 3) and casein specificity (Table 2) with respect to* A. oryzae* ICFC 8/12 (control strain), we decided to explore the potentiality of the* A. sojae* ATCC 20235 proteases for the production of antioxidant peptides from the hydrolysis of casein or gelatin. As shown in Figure 6(a) the degree of hydrolysis (DH) of casein was significantly higher than gelatin after fifteen minutes of hydrolysis reaction, reaching 95% of hydrolysis degree at 120 min. Similar degree of hydrolysis values has been obtained for casein hydrolysis using an immobilized* A. oryzae* protease [42].

Likewise, the antioxidant activity was higher for casein hydrolysates than for gelatin hydrolysates obtained throughout all the hydrolysis time. However the major increase rate was registered for gelatin, possibly because of the rapid increase of small peptides production that ranged between 11 and 25 kDa (Figure 6(b) and Supplementary Data 6). In this context it is important to mention that the initial higher DPPH radical-scavenging activity and the low increase rate observed for casein are due to partial hydrolysis (small peptides) present in the substrate at the initial time (Supplementary Data 6).

## 4. Conclusions

Based on the results obtained in this study we were able to find new* Aspergillus* species and strains with the ability of achieving high and intermediate levels of proteolytic activity. This is the first report for protease production under solid state fermentation for the strains* A. oryzae* NRRL 2217,* A. flavipes* NRRL 295,* A. oryzae* ICFC 8/12,* A. giganteus* NRRL 10,* A. rhizopodus* NRRL 6136,* A. sojae* NRRL 5595, and* A. sojae* ATCC 20235. It was determined that these strains exhibited maximum protease productivity within a short fermentation time using wheat bran as substrate, which significantly reduces the production costs. It is important to note that the crude extracts from* A. sojae* ATCC 20235,* A. oryzae* ICFC 8/12,* A. flavipes* NRRL 295, and* A. giganteus* NRRL 10, which presented the maximum levels of protease productivity, showed remarkable proteolytic activity in a wide range of pH and temperature. The extracts of* A. flavipes* NRRL 295 and* A. sojae* ATCC 20235 presented a notable stability under optimum pH and temperature values with a high half-life (240 min). Likewise, these extracts showed a minimal *Q*_10_ and *E*_a_ values for azo-casein hydrolysis. However, the proteolytic extract from* A. sojae* ATCC 20235 presented a high affinity for azo-casein. Finally, this extract is composed of a variety of proteolytic enzymes of different molecular weights and proved to be an appropriate biocatalyst for hydrolysis of casein and gelatin, increasing the antioxidant activities in 35% and 125%, respectively.

In conclusion,* A. sojae* ATCC 20235 is a promising strain for the production of proteolytic enzymes useful for obtaining peptides from food by-products with nutritional and medicinal relevance.

## Supplementary Material

A state of the art of proteases production by *Aspergillus*, moister determination of wheat bran with different water activities at 28°C, Multifactorial ANOVA and clusters analysis of proteolytic activity from different crude extracts, ANOVA of the influence of temperature and pH on the proteolytic activity of 4 strains of *Aspergillus*, Arhenius plots for proteasas produced by *Aspergillus* strains, SDS-PAGE for casein and gelatin hydrolyzed using protease extracts from *A. sojae*, the Michaelis-Menten constant and maximum velocity values for proteasas from *Aspergillus* strains are provided.

## Figures and Tables

**Table 1:** Activation energy (*E*_a_) and *Q*_10_ for azo-casein hydrolysis of the proteolytic extracts from selected *Aspergillus strains*.

Strain	Temperature range (°C)	*E*_a_ (kJ/mol)	*Q*_10_^*∗*^	*R* ^2^
4	30–40	21.82 ± 1.43^†^	1.31	0.98
8	30–50	38.64 ± 3.27	1.61	0.97
9	30–46	31.66 ± 1.87	1.48	0.99
12	30–46	29.28 ± 3.35	1.44	0.97

^*∗*^ *Q*_10_ determined using the average temperature range.

^†^Std. error of linear regression coefficient.

**Table 2:** Kinetics parameters *K*_*m*_ and *V*_Max⁡_ for proteolytic extracts from selected *Aspergillus strains*.

Strain	*V*_Max⁡_	*V*_Max⁡_	*K*_*m*_	*V*_Max⁡_/*K*_*m*_	*V*_Max⁡_/*K*_*m*_	*R* ^2^
	(U/mL)	(U/gds)	(mg/mL)	(U/mg)	(U·mL/mg·gds)	
12	23.38 ± 1.16^†^	233.8 ± 2.1^†^	1.34 ± 0.26^†^	17.45 ± 0.20^†^	174.5 ± 2.0^†^	0.98
9	20.79 ± 0.98	207.9 ± 9.8	1.48 ± 0.26	14.04 ± 0.18	140.4 ± 1.8	0.98
8	15.51 ± 0.66	155.1 ± 6.6	0.86 ± 0.18	18.03 ± 0.22	180.3 ± 2.2	0.98
4	4.46 ± 0.21	44.6 ± 2.1	0.97 ± 0.21	4.59 ± 0.22	45.9 ± 2.2	0.98

^†^Std. error of nonlinear regression coefficient (Michaelis-Menten kinetics fit).

**Table 3:** Inactivation and statistical parameters estimated using a first-order inactivation model (3). Parameter *K* is the inactivation rate constant and *A*_0_ is the initial activity. For strain reference numbers see Section 2.2.

	Strain 4	Strain 8	Strain 9	Strain 12
Enz. extract				
*A*_0_	0.998	1.000	0.999	0.999
*K* (min)	0.003	0.009	0.021	0.003
Half-life (min)	225.000	75.260	32.910	204.000
Std. error				
*A*_0_	0.004	0.003	0.005	0.003
*K*	0.000	0.000	0.001	0.000
*R* ^2^	0.973	0.995	0.994	0.990

**Table 4:** Estimated molecular weights corresponding to the bands with proteolytic activity for each extract.

Sample	Estimate Mw
4	32
	29
	22

8	32
	34
	26

9	92
	85
	33
	23

12	88
	78
	68
	58
	51
	40
	36
	29
	22
	19
