# Supplementary material for: A Comparative Study of New Aspergillus Strains for Proteolytic Enzymes Production by Solid State Fermentation
Source: Enzyme Res. 2016 Feb 16;2016:3016149. doi: 10.1155/2016/3016149 (PMC4771904; doi:10.1155/2016/3016149)

Supplementary data 1

Author	Title	Year
De Castro, R.J.S. et al.	Production and biochemical properties of proteases secreted by ***Aspergillus niger*** under solid state fermentation in response to different agroindustrial substrates	(2014)
De Castro, R.J.S. et al.	Production and biochemical characterization of protease from ***Aspergillus oryzae***: An evaluation of the physical-chemical parameters using agroindustrial wastes as supports	(2014)
De Castro, R.J.S. et al.	Advantages of an acid protease from ***Aspergillus oryzae*** over commercial preparations for production of whey protein hydrolysates with antioxidant activities	(2014)
Abidi, F. et al.	Purification and biochemical characterization of a novel alkaline protease from ***Aspergillus niger***. Use in antioxidant peptides production	(2014)
Li, C. et al.	Production optimization, purification, and characterization of a novel acid protease from a fusant by ***Aspergillus oryzae*** and ***Aspergillus niger***	(2014)
Sukumprasertsri, M. et al.	Fuzzy logic control of rotating drum bioreactor for improved production of amylase and protease enzymes by ***Aspergillus oryzae*** in solid-state fermentation.	(2013)
Castro-Ochoa, D. et al.	Evaluation of strategies to improve the production of alkaline protease PrtA from **Aspergillus nidulans**	(2013)
Belmessikh, A. et al.	Statistical optimization of culture medium for neutral protease production by ***Aspergillus oryzae***. Comparative study between solid and submerged fermentations on tomato pomace	(2013)
Niyonzima, F.N. et al.	Screening and optimization of cultural parameters for an alkaline protease production by ***Aspergillus terreus*** Gr. under submerged fermentation	(2013)
Rodrigues da Silva, R. et al.	Production and partial characterization of serine and metallo peptidases secreted by ***Aspergillus fumigatus*** Fresenius in submerged and solid state fermentation	(2013)
Dhingra, S. et al.	VeA regulates conidiation, gliotoxin production, and protease activity in the opportunistic human pathogen ***Aspergillus fumigatus***	(2012)
Roja Rani, M. et al.	Screening and selection of ***Aspergillus flavus*** strain for alkaline protease production by submerged fermentation	(2012)
Siala, R. et al.	Optimization of acid protease production by ***Aspergillus niger*** I1 on shrimp peptone using statistical experimental design	(2012)
Lashari, S. et al.	Optimization of culture condition for protease production by ***Aspergillus niger***	(2011)
Castro, A.M. et al.	Multiresponse optimization of inoculum conditions for the production of amylases and proteases by ***Aspergillus awamori*** in solid-state fermentation of babassu cake	(2011)
Leng, X.-W. et al.	Improvement of acid protease production by a mixed culture of ***Aspergillus niger*** and ***Aspergillus oryzae*** using solid-state fermentation technique	(2011)
Kumura, H. et al.	Production and partial purification of proteases from ***Aspergillus oryzae*** grown in a medium based on whey protein as an exclusive nitrogen source	(2011)
Morya, V.K. et al.	Production and partial characterization of neutral protease by an indigenously isolated strain of ***Aspergillus tubingensis*** NIICC-08155	(2010)
Chellapandi, P.	Production and preliminary characterization of alkaline protease from ***Aspergillus flavus*** and ***Aspergillus terreus***	(2010)

Supplementary data 1

Author	Title	Year
Vishwanatha, K.S. et al.	Acid protease production by solid-state fermentation using ***Aspergillus oryzae*** MTCC 5341: Optimization of process parameters	(2010)
Bhatnagar, D. et al.	Amylase and acid protease production by solid state fermentation using ***Aspergillus niger*** from mangrove swamp	(2010)
Hwang, J.Y. et al.	Optimal conditions for the production of salt-tolerant protease from ***Aspergillus sp.*** 101 and its characteristics	(2009)
Mukhtar, H. et al.	Production of acid protease by ***Aspergillus niger*** using solid state fermentation	(2009)
Rajmalwar, S. et al.	Production of protease by ***Aspergillus sp.*** using solid-state fermentation	(2009)
Chutmanop, J. et al.	Protease production by ***Aspergillus oryzae*** in solid-state fermentation using agroindustrial substrates	(2008)
Hajji, M. et al.	Optimization of alkaline protease production by ***Aspergillus clavatus*** ES1 in Mirabilis jalapa tuber powder using statistical experimental design	(2008)
Basu, B.R. et al.	Production and characterization of extracellular protease of mutant ***Aspergillus niger*** AB100 grown on fish scale	(2008)
Anandan, D. et al.	Isolation, characterization and optimization of culture parameters for production of an alkaline protease isolated from ***Aspergillus tamarii***	(2007)
Srinu Babu, G. et al.	Optimization of protease production from ***Aspergillus oryzae*** sp. using Box-Behnken experimental design	(2007)
Srinubabu, G. et al.	Screening of nutritional parameters for the production of protease from ***Aspergillus oryzae***	(2007)
Wu, T.Y. et al.	Investigations on protease production by a wild-type ***Aspergillus terreus*** strain using diluted retentate of pre-filtered palm oil mill effluent (POME) as substrate	(2006)
Negi, S. et al.	Optimization of amylase and protease production from ***Aspergillus awamori*** in single bioreactor through EVOP factorial design technique	(2006)
Sunil Kumar, O. et al.	Studies on cultural conditions and nutritional parameters for the production of protease enzyme by ***Aspergillus oryzae***	(2006)
Ramanathan, T. et al.	Alkaline protease production and optimization in estuary isolate of ***Aspergillus sp***	(2005)
Te Biesebeke, R. et al.	Branching mutants of ***Aspergillus oryzae*** with improved amylase and protease production on solid substrates	(2005)
Sandhya, C. et al.	Comparative evaluation of neutral protease production by ***Aspergillus oryzae*** in submerged and solid-state fermentation	(2005)
Tremacoldi, C.R. et al.	Production of extracellular alkaline proteases by ***Aspergillus clavatus***	(2005)
Wang, R. et al.	Protease production and conidiation by ***Aspergillus oryzae*** in flour fermentation	(2005)
Tremacoldi, C.R. et al.	Production of extracellular acid proteases by ***Aspergillus clavatus***	(2004)

Supplementary data 1

Author	Title	Year
Nehra, K.S. et al.	Production and characterization of alkaline protease from ***Aspergillus sp.*** and its compatibility with commercial detergents	(2004)
Hara, Y. et al.	The effect of electrolyzed water on production of soybean functional low-molecular weight peptide by an ***Aspergillus oryzae*** protease	(2003)
Papagianni, M. et al.	Comparative studies on extracellular protease secretion and glucoamylase production by free and immobilized ***Aspergillus niger*** cultures	(2002)
Aguilar, C.N. et al.	Culture conditions dictate protease and tannase production in submerged and solid-state cultures of ***Aspergillus niger*** Aa-20	(2002)
Nehra, K.S. et al.	Production of alkaline protease by ***Aspergillus sp.*** under submerged and solid substrate fermentation	(2002)
Boer, C.G. et al.	Production of extracellular protease by ***Aspergillus tamarii***	(2000)
Samarntarn, W. et al.	Production of alkaline protease by a genetically engineered ***Aspergillus oryzae*** U1521	(1999)
Mulimani, V.H. et al..	Production of protease by ***Aspergillus flavus*** under solid state fermentation	(1999)
Channe, P.S. et al.	Continuous production of cheese by immobilized milk-clotting protease from ***Aspergillus niger*** MC4	(1998)
Yang, F.-C. et al.	Production of acid protease using thin stillage from a rice-spirit distillery by ***Aspergillus niger***	(1998)
Nehra, K.S. et al.	Production of Alkaline Protease by Immobilized **Aspergillus** Mycelia	(1998)
Yang, Y.-K. et al.	The hybrid formation between ***Aspergillus oryzae*** var. oryzae and Penicillium chrysogenum by nuclear transfer and the production of alkaline protease	(1998)
Sapunova, L.I. et al.	Conditions of synthesis of pectinases and proteases by ***Aspergillus alliaceus*** and production of a complex macerating preparation	(1997)
Taragano, V. et al.	Combined effect of water activity depression and glucose addition on pectinases and protease production by ***Aspergillus niger***	(1997)
Ogawa, A. et al.	Production of neutral protease by membrane-surface liquid culture of ***Aspergillus oryzae*** IAM2704	(1995)
Yasuhara, A. et al..	Production of neutral protease from ***Aspergillus oryzae*** by a novel cultivation method on a microporous membrane	(1994)
Singh, A. et al.	Production of thermostable acid protease by ***Aspergillus niger***	(1994)
Battaglino, R.A. et al.	Culture requirements for the production of protease by ***Aspergillus oryzae*** in solid state fermentation	(1991)
Malathi, S. et al.	Production of alkaline protease by a new ***Aspergillus flavus*** isolate under solid-substrate fermentation conditions for use as a depilation agent	(1991)
Fukushima, Y. et al.	Stimulation of protease production by ***Aspergillus oryzae*** with oils in continuous culture	(1991)

Supplementary data 1

Author	Title	Year
Fukushima, Y. et al.	Continuous protease production in a carbon-limited chemostat culture by salt tolerant ***Aspergillus oryzae***	(1989)
Pourrat, H. et al.	Production of semi-alkaline protease by ***Aspergillus niger***	(1988)
Singh, D.P. et al.	Effect of pH, temperature, nitrogen source and glucose concentrations on acid protease production by ***Aspergillus niger*** mutant	(1975)
Barwald, G. et al.	Microbiologic production of a protease with a fibrinolytic action on ***Aspergillus ochraceus***	(1974)

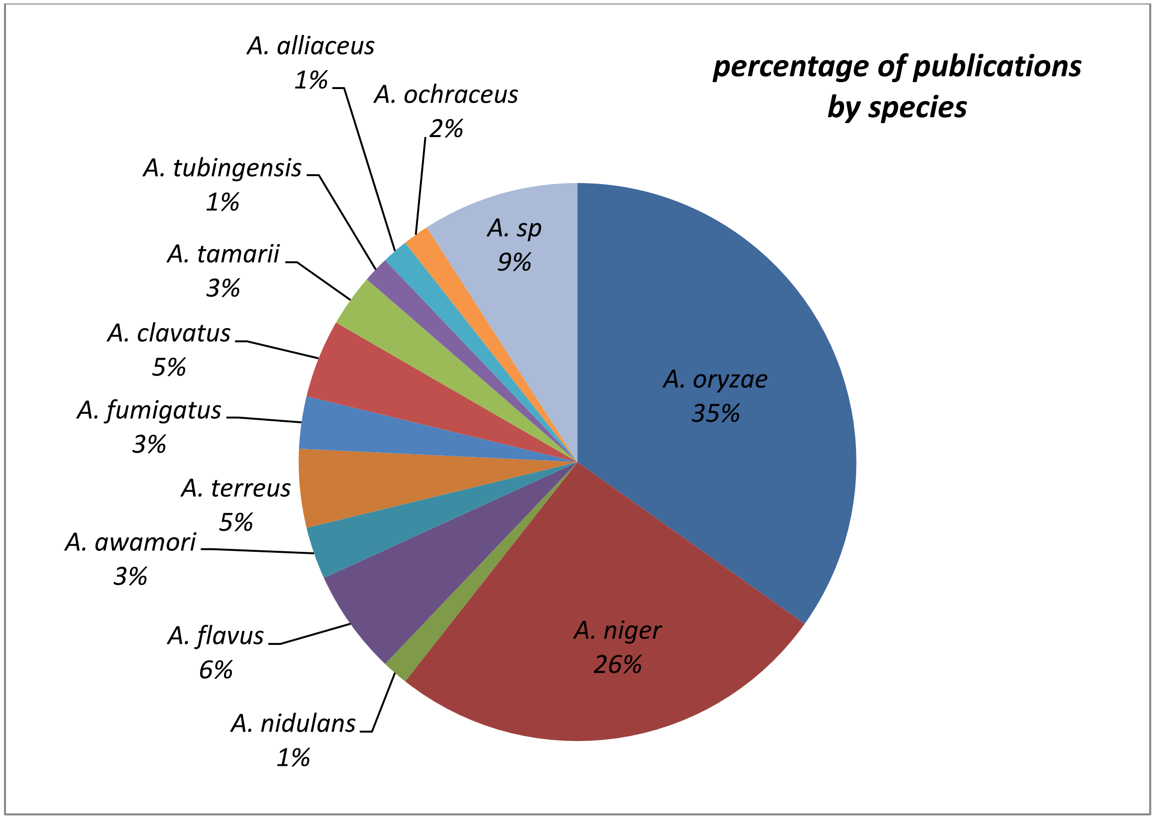

Supplementary data 2

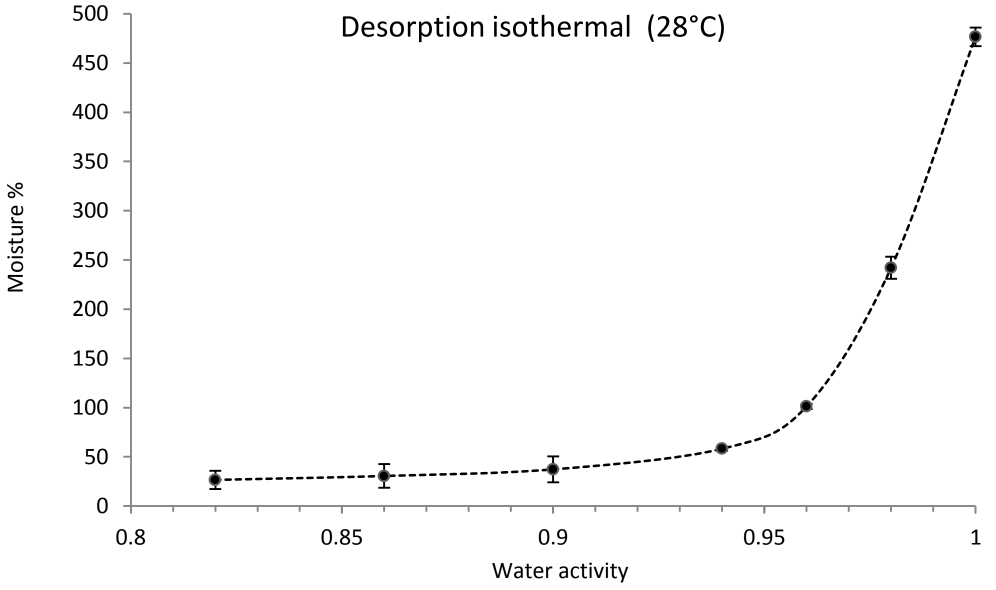

Determination of water activity

For water activity determination, petri plate containing 3g of wheat bran wetted with Czapek-Dox at the maximum absorption capacity (moisture 475%) were placed jointly with different glycerol standard solutions in a hermetic chamber. After preparation, the system was incubated at 28°C for 7 day and the residual moisture was determined by gravimetric measure.

Glycerol standard solution

Water activity	Water (g)	Glycerol (g)
1	25	0.00
0.98	23	2.40
0.96	21	4.48
0.94	19	6.21
0.9	16	9.10
0.86	14	11.66
0.82	12	13.48
0.7	8	17.54

Maximum absorption capacity

The maximum absorption capacity was performed according (Soares de Castro and Sato, 2014a)

Supplementary data 3

Multifactor ANOVA-Hydrolysis Index (HI)

Dependent variable: HI
Factors:
A.Strain *(Asperguillus strins)*
pH
Day

Number of complete cases: 144

This procedure performs a multifactor analysis of variance for HI. It constructs various tests and graphs to determine which factors have a statistically significant effect on HI. It also tests for significant interactions amongst the factors, given sufficient data. The F-tests in the ANOVA table will allow you to identify the significant factors. For each significant factor, the Multiple Range Tests will tell you which means are significantly different from which others.

Analysis of Variance(ANOVA) for HI - Type III Sums of Squares

Source	*Sum of Squares*	*Df*	*Mean Square*	*F-Ratio*	*P-Value*
MAIN EFFECTS					
A:A.Strain	94.7878	11	8.61707	71.33	0.0000
B:pH	6.98286	3	2.32762	19.27	0.0000
C:Day	2.05265	2	1.02633	8.50	0.0003
RESIDUAL	15.3421	127	0.120804		
TOTAL (CORRECTED)	119.165	143			

All F-ratios are based on the residual mean square error.

The ANOVA table decomposes the variability of HI into contributions due to various factors. Since Type III sums of squares have been chosen, the contribution of each factor is measured having removed the effects of all other factors. The P-values test the statistical significance of each of the factors. Since 3 P-values are less than 0.05, these factors have a statistically significant effect on HI at the 95.0% confidence level.

Table of Least Squares Means for HI with 95.0% Confidence Intervals

			Stnd.	*Lower*	*Upper*
Level	*Count*	*Mean*	*Error*	*Limit*	*Limit*
GRAND MEAN	144	1.42549			
A.Strain					
1	12	0.0	0.100334	-0.198544	0.198544
2	12	2.1775	0.100334	1.97896	2.37604
3	12	1.20167	0.100334	1.00312	1.40021
4	12	2.07917	0.100334	1.88062	2.27771
5	12	1.0525	0.100334	0.853956	1.25104
6	12	0.263333	0.100334	0.0647893	0.461877
7	12	0.249167	0.100334	0.0506226	0.447711
8	12	2.185	0.100334	1.98646	2.38354
9	12	1.96167	0.100334	1.76312	2.16021
10	12	1.76583	0.100334	1.56729	1.96438
11	12	1.86083	0.100334	1.66229	2.05938
12	12	2.30917	0.100334	2.11062	2.50771
pH					
6	36	1.80583	0.0579281	1.6912	1.92046
7	36	1.31778	0.0579281	1.20315	1.43241
8	36	1.27278	0.0579281	1.15815	1.38741
9	36	1.30556	0.0579281	1.19093	1.42019
Day					
2	48	1.25667	0.0501672	1.15739	1.35594
4	48	1.50729	0.0501672	1.40802	1.60656
6	48	1.5125	0.0501672	1.41323	1.61177

This table shows the mean HI for each level of the factors. It also shows the standard error of each mean, which is a measure of its sampling variability. The rightmost two columns show 95.0% confidence intervals for each of the means.

Multiple Range Tests for HI by A.Strain

Method: 95.0 percent LSD

A.Strain	*Count*	*LS Mean*	*LS Sigma*	*Homogeneous Groups*
1	12	0.0	0.100334	X
7	12	0.249167	0.100334	X
6	12	0.263333	0.100334	X
5	12	1.0525	0.100334	X
3	12	1.20167	0.100334	X
10	12	1.76583	0.100334	X
11	12	1.86083	0.100334	XX
9	12	1.96167	0.100334	XXX
4	12	2.07917	0.100334	XXX
2	12	2.1775	0.100334	XX
8	12	2.185	0.100334	XX
12	12	2.30917	0.100334	X

Contrast	*Sig.*	*Difference*	*+/- Limits*
1 - 2	*	-2.1775	0.280784
1 - 3	*	-1.20167	0.280784
1 - 4	*	-2.07917	0.280784
1 - 5	*	-1.0525	0.280784
1 - 6		-0.263333	0.280784
1 - 7		-0.249167	0.280784
1 - 8	*	-2.185	0.280784
1 - 9	*	-1.96167	0.280784
1 - 10	*	-1.76583	0.280784
1 - 11	*	-1.86083	0.280784
1 - 12	*	-2.30917	0.280784
2 - 3	*	0.975833	0.280784
2 - 4		0.0983333	0.280784
2 - 5	*	1.125	0.280784
2 - 6	*	1.91417	0.280784
2 - 7	*	1.92833	0.280784
2 - 8		-0.0075	0.280784
2 - 9		0.215833	0.280784
2 - 10	*	0.411667	0.280784
2 - 11	*	0.316667	0.280784
2 - 12		-0.131667	0.280784
3 - 4	*	-0.8775	0.280784
3 - 5		0.149167	0.280784
3 - 6	*	0.938333	0.280784
3 - 7	*	0.9525	0.280784
3 - 8	*	-0.983333	0.280784
3 - 9	*	-0.76	0.280784
3 - 10	*	-0.564167	0.280784
3 - 11	*	-0.659167	0.280784
3 - 12	*	-1.1075	0.280784
4 - 5	*	1.02667	0.280784
4 - 6	*	1.81583	0.280784
4 - 7	*	1.83	0.280784
4 - 8		-0.105833	0.280784
4 - 9		0.1175	0.280784
4 - 10	*	0.313333	0.280784
4 - 11		0.218333	0.280784
4 - 12		-0.23	0.280784
5 - 6	*	0.789167	0.280784

5 - 7	*	0.803333	0.280784
5 - 8	*	-1.1325	0.280784
5 - 9	*	-0.909167	0.280784
5 - 10	*	-0.713333	0.280784
5 - 11	*	-0.808333	0.280784
5 - 12	*	-1.25667	0.280784
6 - 7		0.0141667	0.280784
6 - 8	*	-1.92167	0.280784
6 - 9	*	-1.69833	0.280784
6 - 10	*	-1.5025	0.280784
6 - 11	*	-1.5975	0.280784
6 - 12	*	-2.04583	0.280784
7 - 8	*	-1.93583	0.280784
7 - 9	*	-1.7125	0.280784
7 - 10	*	-1.51667	0.280784
7 - 11	*	-1.61167	0.280784
7 - 12	*	-2.06	0.280784
8 - 9		0.223333	0.280784
8 - 10	*	0.419167	0.280784
8 - 11	*	0.324167	0.280784
8 - 12		-0.124167	0.280784
9 - 10		0.195833	0.280784
9 - 11		0.100833	0.280784
9 - 12	*	-0.3475	0.280784
10 - 11		-0.095	0.280784
10 - 12	*	-0.543333	0.280784
11 - 12	*	-0.448333	0.280784

* denotes a statistically significant difference.

This table applies a multiple comparison procedure to determine which means are significantly different from which others. The bottom half of the output shows the estimated difference between each pair of means. An asterisk has been placed next to 49 pairs, indicating that these pairs show statistically significant differences at the 95.0% confidence level. At the top of the page, 6 homogenous groups are identified using columns of X's. Within each column, the levels containing X's form a group of means within which there are no statistically significant differences. The method currently being used to discriminate among the means is Fisher's least significant difference (LSD) procedure. With this method, there is a 5.0% risk of calling each pair of means significantly different when the actual difference equals 0.

Proteolytic extract from strain 4

Design of experiment matrix and activity results

Experiment	pH	Temperature °C	Activity (U/mL)	Activity (U/mL)
1	6.0	30.0	17.17	20.17
2	8.0	30.0	23.50	27.25
3	6.0	50.0	24.00	23.83
4	8.0	50.0	14.33	14.83
5	5.6	40.0	22.25	21.92
6	8.4	40.0	29.58	27.67
7	7.0	25.9	15.83	17.25
8	7.0	54.1	8.42	16.75
9	7.0	40.0	28.75	32.00
10	7.0	40.0	27.67	25.17
11	7.0	40.0	27.67	25.25

Analysis of variance for activity

Source	*Sum of Squares*	*Df*	*Mean Square*	*F-Ratio*	*P-Value*
A:pH	10.9526	1	10.9526	1.75	0.2083
B:Temperature	31.0428	1	31.0428	4.97	0.0441
AA	11.965	1	11.965	1.91	0.1897
AB	128.641	1	128.641	20.59	0.0006
BB	466.304	1	466.304	74.62	0.0000
Lack-of-fit	37.1071	3	12.369	1.98	0.1669
Pure error	81.2335	13	6.24873		
Total (corr.)	764.437	21			

R-squared = 84.5192 percent
R-squared (adjusted for d.f.) = 79.6815 percent
Standard Error of Est. = 2.49975
Mean absolute error = 1.90762
Durbin-Watson statistic = 1.93975 (P=0.4634)
Lag 1 residual autocorrelation = 0.00325524

The ANOVA table partitions the variability in Activity into separate pieces for each of the effects. It then tests the statistical significance of each effect by comparing the mean square against an estimate of the experimental error. In this case, 3 effects have P-values less than 0.05, indicating that they are significantly different from zero at the 95.0% confidence level.

The lack of fit test is designed to determine whether the selected model is adequate to describe the observed data, or whether a more complicated model should be used. The test is performed by comparing the variability of the current model residuals to the variability between observations at replicate settings of the factors. Since the P-value for lack-of-fit in the ANOVA table is greater or equal to 0.05, the model appears to be adequate for the observed data at the 95.0% confidence level.

The R-Squared statistic indicates that the model as fitted explains 84.5192% of the variability in Activity. The adjusted R-squared statistic, which is more suitable for comparing models with different numbers of independent variables, is 79.6815%. The standard error of the estimate shows the standard deviation of the residuals to be 2.49975. The mean absolute error (MAE) of 1.90762 is the average value of the residuals. The Durbin-Watson (DW) statistic tests the residuals to determine if there is any significant correlation based on the order in which they occur in your data file. Since the P-value is greater than 5.0%, there is no indication of serial autocorrelation in the residuals at the 5.0% significance level.

Supplementary data 4

Model and regression coeffs. for activity

Coefficient	*Estimate*
Constant	-237.991
A:pH	31.2773
B:Temperature	6.00624
AA	-1.02928
AB	-0.308462
BB	-0.0380208

This panel displays the regression equation which has been fitted to the data. The equation of the fitted model is

Activity = -237.991 + 31.2773*pH + 6.00624*Temperature - 1.02928*pH^2 - 0.308462*pH*Temperature - 0.0380208*Temperature^2

Where the values of the variables are specified in their original units.

Optimized response

Optimum value = 28.8046 (U/mL)

Factor	*Low*	*High*	*Optimum*
pH	5.58579	8.41421	8.41421
Temperature	33.6152	70.3848	44.8533

This table shows the combination of factor levels which maximizes activity over the indicated region.

Models validation experiments

Trial	pH	Temperature °C	Experimental (U/mL)	Predicted (U/mL)
1	8.4	25.8	13.8	15.0
2	8.4	33.6	18.8	23.9
3	8.4	40	25.3	27.9
Pearson correlation coefficient (R)			0.96	

Proteolytic extract from strain 8

Design of experiment matrix and activity results

Experiment	pH	Temperature °C	Activity (U/mL)	Activity (U/mL)
1	6.0	39.0	43.1	37.9
2	8.0	39.0	33.9	30.4
3	6.0	65.0	11.33*	10.08*
4	8.0	65.0	5.9	6.3
5	5.6	52.0	47.0	47.5
6	8.4	52.0	13.8	12.9
7	7.0	33.6	27.8	25.3
8	7.0	70.4	6.7	5.8
9	7.0	52.0	47.0	45.9
10	7.0	52.0	44.2	40.9
11	7.0	52.0	44.2	41.5

* Outliers points

Analysis of variance for activity

Source	*Sum of Squares*	*Df*	*Mean Square*	*F-Ratio*	*P-Value*
A:pH	1282.77	1	1282.77	254.85	0.0001
B:Temperature	731.405	1	731.405	145.31	0.0003
AA+block	341.196	1	341.196	67.79	0.0012
AB	100.295	1	100.295	19.93	0.0111
BB+block	1699.45	1	1699.45	337.64	0.0001
Blocks	18.432	1	18.432	3.66	0.1282
Lack-of-fit	150.287	9	16.6986	3.32	0.1301
Pure error	20.1333	4	5.03333		
Total (corr.)	4890.4	19			

R-squared = 96.5152 percent
R-squared (adjusted for d.f.) = 94.9068 percent
Standard Error of Est. = 2.24351
Mean absolute error = 2.50531
Durbin-Watson statistic = 1.05564 (P=0.0115)
Lag 1 residual autocorrelation = 0.440936

The ANOVA table partitions the variability in Activity into separate pieces for each of the effects. It then tests the statistical significance of each effect by comparing the mean square against an estimate of the experimental error. In this case, 5 effects have P-values less than 0.05, indicating that they are significantly different from zero at the 95.0% confidence level.

The lack of fit test is designed to determine whether the selected model is adequate to describe the observed data, or whether a more complicated model should be used. The test is performed by comparing the variability of the current model residuals to the variability between observations at replicate settings of the factors. Since the P-value for lack-of-fit in the ANOVA table is greater or equal to 0.05, the model appears to be adequate for the observed data at the 95.0% confidence level.

The R-Squared statistic indicates that the model as fitted explains 96.5152% of the variability in Activity. The adjusted R-squared statistic, which is more suitable for comparing models with different numbers of independent variables, is 94.9068%. The standard error of the estimate shows the standard deviation of the residuals to be 2.24351. The mean absolute error (MAE) of 2.50531 is the average value of the residuals. The Durbin-Watson (DW) statistic tests the residuals to determine if there is any significant correlation based on the order in which they occur in your data file. Since the P-value is less than 5.0%, there is an indication of possible serial correlation at the 5.0% significance level.

Model and regression coeffs. for activity

Regression coeffs. for Activity

Coefficient	*Estimate*
Constant	-463.439
A:pH	88.4276
B:Temperature	9.61254
AA	-5.75234
AB	-0.352064
BB	-0.0745077

The StatAdvisor
This pane displays the regression equation which has been fitted to the data. The equation of the fitted model is

Activity = -463.439 + 88.4276*pH + 9.61254*Temperature - 5.75234*pH^2 - 0.352064*pH*Temperature - 0.0745077*Temperature^2

Where the values of the variables are specified in their original units.

Optimized response

Optimum value = 48.921 (U/mL)

Factor	*Low*	*High*	*Optimum*
pH	5.6	8.4	6.15718
Temperature	25.9	54.1	49.9589

This table shows the combination of factor levels which maximizes activity over the indicated region.

Models validation experiments

Trial	pH	Temperature °C	Experimental (U/mL)	Predicted (U/mL)
1	6.4	29.5	29.5	30.1
2	6.4	51.6	51.6	48.1
3	6.4	70.4	6.9	15.7
Pearson correlation coefficient (R)			0.99	

Supplementary data 4

Proteolytic extract from strain 9

Design of experiment matrix and activity results

Experiment	Ph	Temperature °C	Activity (U/mL)	Activity (U/mL)
1	6.0	30.0	29.67	27.00
2	8.0	30.0	17.50	20.42
3	6.0	50.0	49.75*	48.25
4	8.0	50.0	9.08	9.33
5	5.6	40.0	43.92	43.83
6	8.4	40.0	15.67	17.42
7	7.0	25.9	15.58	16.42
8	7.0	54.1	15.50	17.42
9	7.0	40.0	39.17	37.67
10	7.0	40.0	37.75	37.67
11	7.0	40.0	36.33	33.83

* Outliers points

Analysis of variance for activity

Source	*Sum of Squares*	*Df*	*Mean Square*	*F-Ratio*	*P-Value*
A:pH	866.961	1	866.961	250.15	0.0001
B:Temperature	839.184	1	839.184	242.13	0.0001
AA+block	91.4708	1	91.4708	26.39	0.0068
AB	258.081	1	258.081	74.47	0.0010
BB+block	1074.68	1	1074.68	310.08	0.0001
Blocks	1.01154	1	1.01154	0.29	0.6177
Lack-of-fit	71.0904	9	7.89893	2.28	0.2220
Pure error	13.8632	4	3.4658		
Total (corr.)	2774.29	19			

R-squared = 96.9378 percent
R-squared (adjusted for d.f.) = 95.5245 percent
Standard Error of Est. = 1.86167
Mean absolute error = 1.56887
Durbin-Watson statistic = 2.46733 (P=0.7725)
Lag 1 residual autocorrelation = -0.35041

The StatAdvisor
The ANOVA table partitions the variability in Activity into separate pieces for each of the effects. It then tests the statistical significance of each effect by comparing the mean square against an estimate of the experimental error. In this case, 5 effects have P-values less than 0.05, indicating that they are significantly different from zero at the 95.0% confidence level.

The lack of fit test is designed to determine whether the selected model is adequate to describe the observed data, or whether a more complicated model should be used. The test is performed by comparing the variability of the current model residuals to the variability between observations at replicate settings of the factors. Since the P-value for lack-of-fit in the ANOVA table is greater or equal to 0.05, the model appears to be adequate for the observed data at the 95.0% confidence level.

The R-Squared statistic indicates that the model as fitted explains 96.9378% of the variability in Activity. The adjusted R-squared statistic, which is more suitable for comparing models with different numbers of independent variables, is 95.5245%. The standard error of the estimate shows the standard deviation of the residuals to be 1.86167. The mean absolute error (MAE) of 1.56887 is the average value of the residuals. The Durbin-Watson (DW) statistic tests the residuals to determine if there is any significant correlation based on the order in which they occur in your data file. Since

the P-value is greater than 5.0%, there is no indication of serial autocorrelation in the residuals at the 5.0% significance level.

Model and regression coeffs. for activity

Coefficient	*Estimate*
Constant	-387.131
A:pH	57.9181
B:Temperature	12.8527
AA	-2.93254
AB	-0.68845
BB	-0.0993071

This panel displays the regression equation which has been fitted to the data. The equation of the fitted model is:

Activity = -387.131 + 57.9181*pH + 12.8527*Temperature - 2.93254*pH^2 - 0.68845*pH*Temperature - 0.0993071*Temperature^2

Where the values of the variables are specified in their original units.

Optimized response

Optimum value = 49.0394 (U/mL)

Factor	*Low*	*High*	*Optimum*
pH	5.6	8.4	5.6
Temperature	25.9	54.1	45.3051

This table shows the combination of factor levels which maximizes activity over the indicated region.

Models validation experiments

Trial	pH	Temperature °C	Experimental (U/mL)	Predicted (U/mL)
1	5.6	25.8	13.3	11.5
2	5.6	33.6	24.6	35.5
3	5.6	40	32.0	46.2
Pearson correlation coefficient (R)			0.99	

Supplementary data 4

Proteolytic extract from strain 12

Design of experiment matrix and activity results

Experiment	pH	Temperature °C	Activity (U/mL)	Activity (U/mL)
1	6.0	39.0	56.8	60.8
2	8.0	39.0	39.1	33.4
3	6.0	65.0	15.1*	15.3*
4	8.0	65.0	6.4	6.5
5	5.6	52.0	64.2	59.4
6	8.4	52.0	27.7	27.7
7	7.0	33.6	33.0	32.6
8	7.0	70.4	9.8	10.5
9	7.0	52.0	66.1	63.3
10	7.0	52.0	56.8	61.7
11	7.0	52.0	56.8	59.6

* Outliers points

Analysis of Variance for Activity

Source	*Sum of Squares*	*Df*	*Mean Square*	*F-Ratio*	*P-Value*
A:pH	2031.41	1	2031.41	125.89	0.0004
B:Temperature	1219.03	1	1219.03	75.54	0.0010
AA+block	583.995	1	583.995	36.19	0.0038
AB	35.8033	1	35.8033	2.22	0.2106
BB+block	3867.01	1	3867.01	239.64	0.0001
blocks	0.072	1	0.072	0.00	0.9499
Lack-of-fit	145.112	9	16.1235	1.00	0.5446
Pure error	64.5467	4	16.1367		
Total (corr.)	8752.88	19			

R-squared = 97.6047 percent
R-squared (adjusted for d.f.) = 96.4992 percent
Standard Error of Est. = 4.01705
Mean absolute error = 2.62582
Durbin-Watson statistic = 2.20327 (P=0.6196)
Lag 1 residual autocorrelation = -0.114815

The StatAdvisor
The ANOVA table partitions the variability in Activity into separate pieces for each of the effects. It then tests the statistical significance of each effect by comparing the mean square against an estimate of the experimental error. In this case, 4 effects have P-values less than 0.05, indicating that they are significantly different from zero at the 95.0% confidence level.

The lack of fit test is designed to determine whether the selected model is adequate to describe the observed data, or whether a more complicated model should be used. The test is performed by comparing the variability of the current model residuals to the variability between observations at replicate settings of the factors. Since the P-value for lack-of-fit in the ANOVA table is greater or equal to 0.05, the model appears to be adequate for the observed data at the 95.0% confidence level.

The R-Squared statistic indicates that the model as fitted explains 97.6047% of the variability in Activity. The adjusted R-squared statistic, which is more suitable for comparing models with different numbers of independent variables, is 96.4992%. The standard error of the estimate shows the standard deviation of the residuals to be 4.01705. The mean absolute error (MAE) of 2.62582 is the average value of the residuals. The Durbin-Watson (DW) statistic tests the residuals to determine if there is any significant correlation based on the order in which they occur in your data file. Since the P-value is greater than 5.0%, there is no indication of serial autocorrelation in the residuals at the 5.0% significance level.

Model and regression coeffs. for activity

Regression coeffs. for Activity

Coefficient	*Estimate*
constant	-556.483
A:pH	103.195
B:Temperature	12.3857
AA	-7.52571
AB	-0.21035
BB	-0.112392

The StatAdvisor
This pane displays the regression equation which has been fitted to the data. The equation of the fitted model is

Activity = -556.483 + 103.195*pH + 12.3857*Temperature - 7.52571*pH^2 - 0.21035*pH*Temperature - 0.112392*Temperature^2

Where the values of the variables are specified in their original units

Optimized response

Optimum value = 67.1965

Factor	*Low*	*High*	*Optimum*
pH	5.6	8.4	6.16687
Temperature	25.9	54.1	49.3299

This table shows the combination of factor levels which maximizes activity over the indicated region.

Models validation experiments

Trial	pH	Temperature °C	Experimental (U/mL)	Predicted (U/mL)
1	6.4	33.6	36.4	39.8
2	6.4	52.0	71.9	65.9
3	6.4	70.4	11.3	15.9
Pearson correlation coefficient (R)			0.99	

Supplementary data 5

Arrhenius Plots

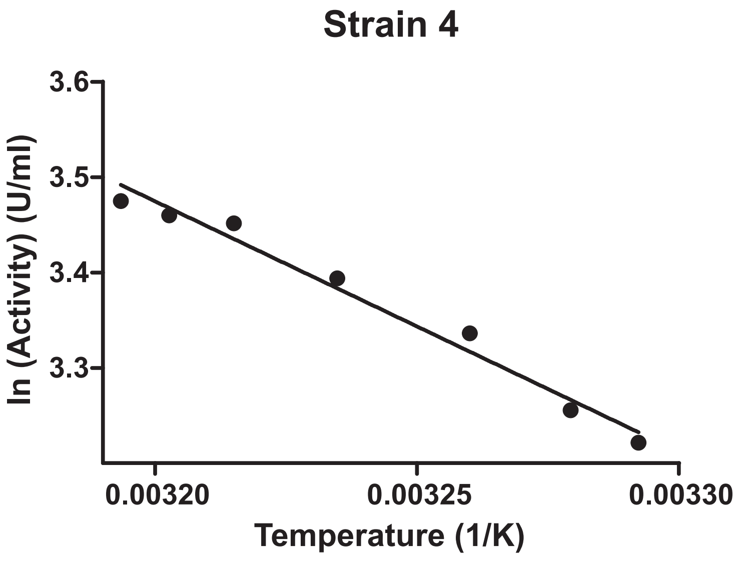

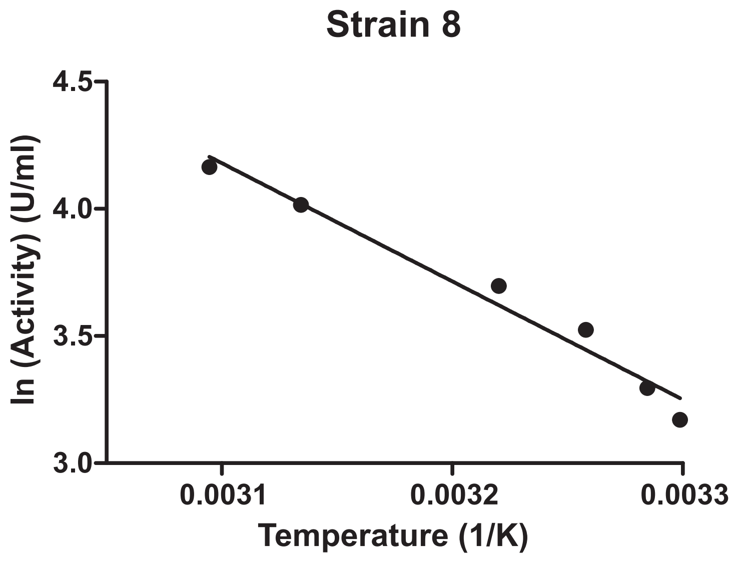

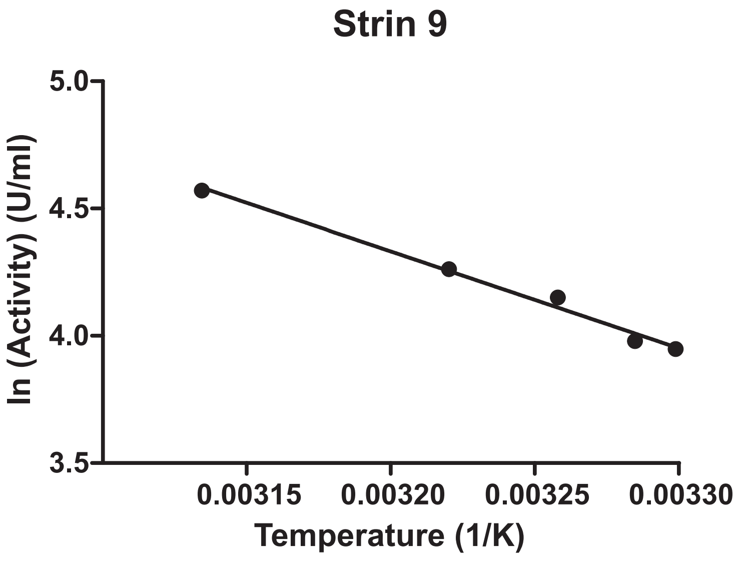

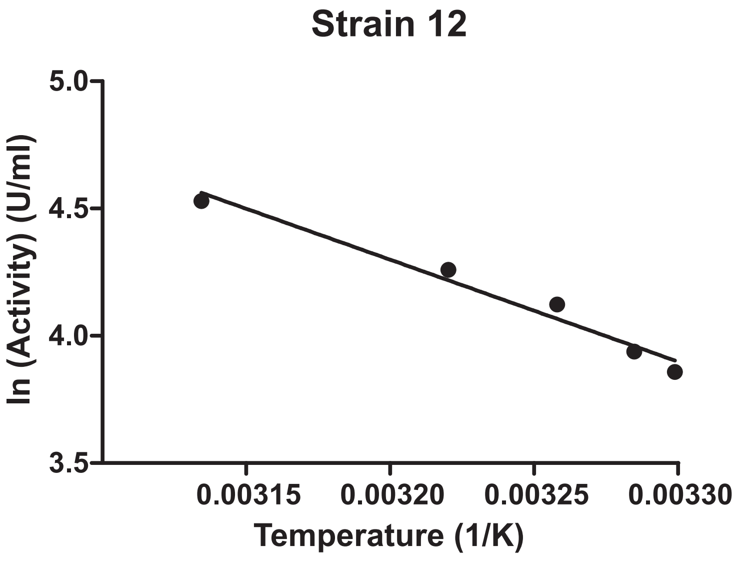

Strain	4	8	9	12
Ea/R	-2624 ± 172.6	-4647 ± 392.9	-3808 ± 224.9	-4006 ± 402.9
lnA	11.87 ± 0.5591	18.59 ± 1.264	16.52 ± 0.7288	17.12 ± 1.305
R^2	0.9788	0.9722	0.9896	0.9705

Supplementary data 6

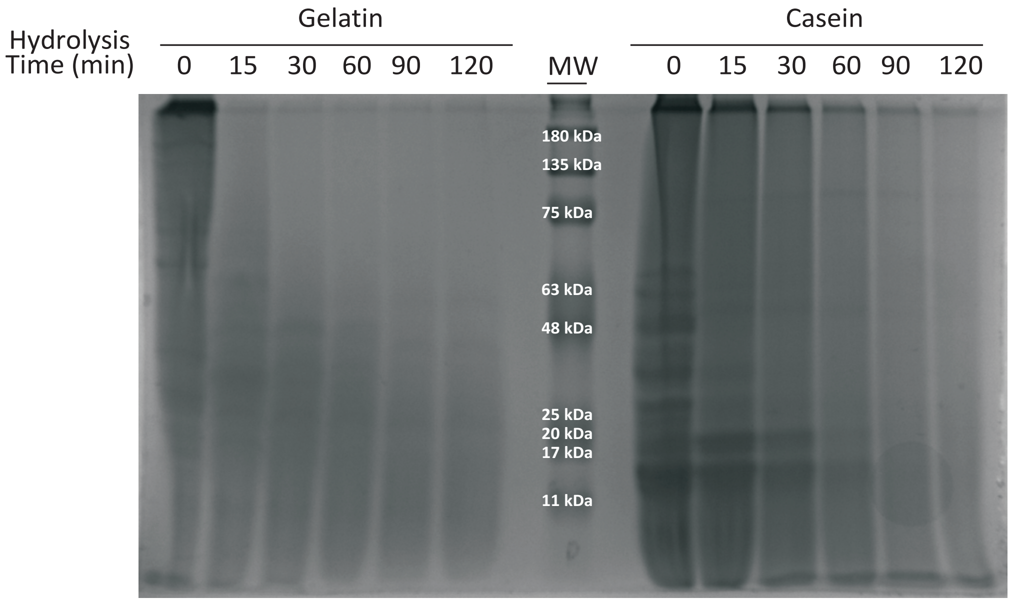

SDS-PAGE for casein and gelatin hydrolyzed using protease extracts from A. sojae. MW: Molecular weight marker Arcoiris PB-L productos Bio-Lógicos®

Supplementary data 7

Michaelis–Menten kinetics

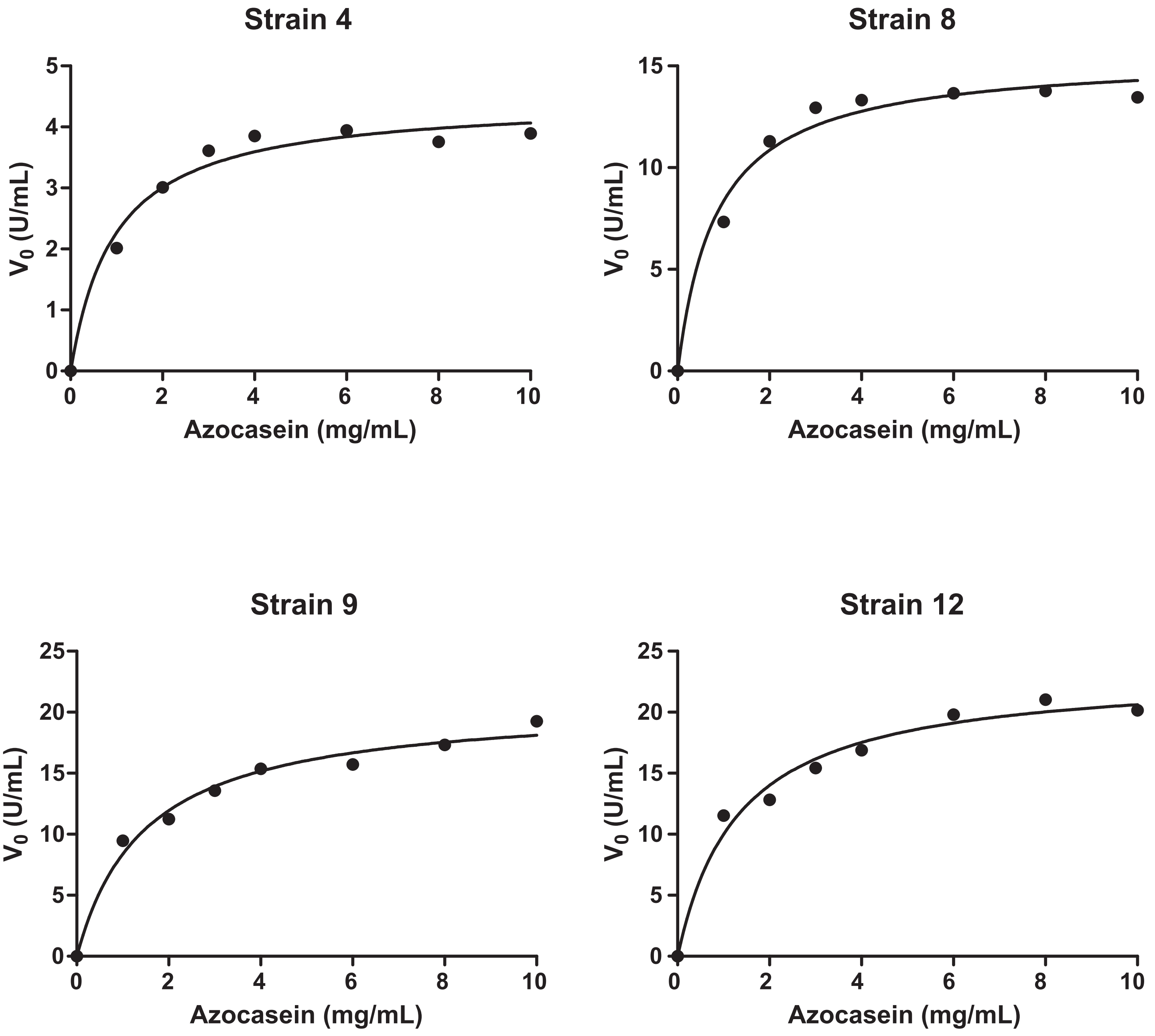

Supplement: Supplementary file 1 — A state of the art of proteases production by Aspergillus, moister determination of wheat bran with different water activities at 28°C, Multifactorial ANOVA and clusters analysis of proteolytic activity from different crude extracts, ANOVA of the influence of temperature and pH on the proteolytic activity of 4 strains of Aspergillus, Arhenius plots for proteasas produced by Aspergillus strains, SDS-PAGE for casein and gelatin hydrolyzed using protease extracts from A. sojae, the Michaelis-Menten constant and maximum velocity values for proteasas from Aspergillus strains are provided. [file 3016149.f1.pdf]
